# Taking the strain? Police well-being in the COVID-19 era

**DOI:** 10.1177/0032258X211044702

**Published:** 2022-03

**Authors:** Geoff Newiss, Sarah Charman, Camille Ilett, Stephanie Bennett, Aram Ghaemmaghami, Paul Smith, Robert Inkpen

**Affiliations:** 171171 University of Portsmouth, Portsmouth, UK

**Keywords:** Well-being, police, safety, working practices, frontline

## Abstract

Drawing on survey and interview data collected in one police force area, this article considers the varied impacts on police well-being arising during the COVID-19 pandemic. Approximately one-third of police officers surveyed reported feeling less safe in their role during the pandemic, and nearly half suffered increased anxiety. The toll on well-being appears to be most acute for frontline officers and those with caring responsibilities, and is strongly associated with increases in workload. The task of ‘repairing’ well-being will require detailed and sensitive consideration involving genuine efforts to hear the voices of those who have endured this prolonged tour of duty.

## Background

Despite being a regularly repeated phrase, there is perhaps more evidence than at other times to suggest that policing and the policing environment are currently undergoing significant change. Relatively, stable crime figures over long periods of this century have hidden important changes to both crime types and the social harms associated with human behaviour (Higgins and Hales, 2016). Policing demand has changed and stretched the service in ways in which were perhaps difficult to predict. Police forces are tackling the investigatory complexities and serious harms associated with child sexual exploitation, domestic and sexual abuse, human trafficking, organised crime and terrorism while at the same time dealing with enormous increases in non–crime-related incidents which account for 83% of all ‘command and control’ calls received (College of Policing, 2015; NAO, 2018). The last decade has seen falling police numbers and declining police budgets; a current Uplift programme to restore these numbers and a transformation of the education and training provision for all new police officers was conducted. These challenges alone have stretched the capacity and capability of police forces, with Her Majesty’s Inspectorate of Constabulary (HMIC) raising a ‘deep red warning flag’ (2017: 4) over the impact of managing such demand.

Amidst this already threatening storm, in March 2020, the World Health Organisation confirmed the COVID-19 disease as a global pandemic and England entered its first national lockdown with the associated ‘stay at home’ messaging to be enforced through the Health Protection (Coronavirus, Restrictions) (England) Regulations 2020. Research considering the impact of the pandemic upon the well-being of citizens has warned of high levels (even clinically high) of suicidal thoughts and mental distress (Dawson and Golijani-Moghaddam, 2020; O’Connor et al., 2020; Sibley et al., 2020).

A focus from policing bodies towards the mental health and well-being of its workforce emerged before the COVID-19 pandemic. Oscar Kilo (the home of the National Wellbeing Service) launched in 2017 and with it the Bluelight Wellbeing Framework which operates as a tool for employer self-assessment (Kilo, nd), both developments with strong support and involvement across national policing bodies. These initiatives built upon emerging concerns emanating from research suggesting that well-being amongst police officers was lower than in other professions (Johnson et al., 2005; MIND, 2016). For example, Police Federation surveys have found around 80% of officers reported feelings of stress, low mood or anxiety with over 90% of these attributing this to work or being exacerbated by work (Elliott-Davies and Houdmont, 2017; Elliott-Davies, 2018). This is in comparison with only 26% of the adult population attributing their difficulties to work (Elliott-Davies, 2018). The prevalence of post-traumatic stress disorder and complex post-traumatic stress disorder amongst UK police officers also shows higher levels than the international average (Brewin et al., 2020). The impact of these feelings of stress, low mood and anxiety can be felt both individually and organisationally. Individually, there is evidence of low levels of emotional energy and high levels of disturbed sleep (Graham et al., 2020), self-blame (Edwards et al., 2021), somatization and low self-esteem (Demou et al., 2020). Organisationally, evidence from 20 police forces in England and Wales from 2008 to 2018 found the numbers of employees absent due to psychological or mental ill health almost doubled from 4.72 to 8.82% with stress representing over half of these cases (Cartwright and Roach, 2020).

A number of factors affect reported levels of well-being. Research indicates that while high levels of support, a sense of belonging, fair and visible leadership and an affirming environment were all positively related to psychological well-being (Birch et al., 2017; Demou et al., 2020; Jackman et al., 2020; Ordon et al., 2019), the impact of rapid change, not feeling valued, heavy workloads and the negative characteristics of organisational cultures conversely had an adverse impact upon well-being (Demou et al., 2020; Hesketh et al., 2017; Purba and Demou, 2019). There is also evidence to suggest that role and rank can be a determinant of well-being with those in higher ranks, police staff and those in force command or operational support (firearms and public order) all displaying higher levels of well-being than other roles and ranks (Demou et al., 2020; Graham et al., 2020; Jackman et al., 2020).

Whilst the COVID-19 pandemic may not have been the catalyst for a focus upon police well-being, it is likely that its associated challenges will ensure its retention as a key area of concern. However, although there is research that uses past crises and disasters to speculate upon the impact of the COVID-19 pandemic upon police well-being (Drew and Martin, 2020; Laufs and Waseem, 2020; Stogner et al., 2020), there is at the time of writing, relatively little evidence about the actual impact of policing during a pandemic on the well-being of police officers. Emerging findings from Crest Advisory (2020) suggest that there are growing concerns about the potentially hidden impact that a combination of pandemic-related policing activities alongside regular policing duties will have on levels of fatigue and well-being amongst police officers. This appears to be supported by the latest welfare survey from the Police Federation which showed 27% of respondents working longer hours and 53% of respondents indicating that fatigue made carrying out work tasks difficult (Elliott-Davies, 2021). Over half of those reporting psychological difficulties attributed this to excessive workloads with 20% indicating the negative relationship between their well-being and policing during a pandemic. Almost a quarter of respondents to the national survey stated that a member of the public had attempted to breathe or cough on them in order to pass on or give the impression of passing on the COVID-19 disease (Elliott-Davies, 2021). These potentially dangerous situations also affect police officer anxiety levels about the effect of infection upon their families (De Camargo, 2021).

It is the intention of this article, therefore, to consider the issues arising from the policing of the pandemic which have impacted upon police officers’ sense of their own well-being, with well-being referring to ‘a state of complete physical, mental and social well-being and not merely the absence of disease or infirmity’ (World Health Organisation, nd: 2). Well-being has been a clear and prominent theme from the results of our major ESRC-funded research project considering the impact of pandemic policing on both police and public. In a collaboration between the University of Portsmouth and Hampshire Constabulary and using surveys, interviews, focus groups, body-worn video footage and video diaries, this research has investigated both public compliance and organisational resilience. The data for this particular article, as will be seen below, will be drawn from a survey and interviews. The findings will attempt to scrutinise both the organisational and operational stressors which have impacted upon the working and personal lives of police officers as they navigate the continually changing demands of pandemic policing. These organisational stressors (e.g. inconsistent or poor supervision or management, bureaucracy or police numbers) and operational stressors (e.g. the nature of the work and the police role) have been shown to have differing effects upon officers with organisational stressors having the more negative impact upon levels of stress and well-being (Brown and Campbell, 1990; Brough, 2004; Charman and Bennett, 2021; Dick, 2011). This article will assess the potential consequences of a damaged sense of well-being and will consider whether the shared expectations and obligations of employee and employer are sufficiently attuned to be able to maintain a sense of organisational commitment.

## Method

A survey was distributed to all Hampshire Constabulary police officers and staff (*N* = 5077) during the summer of 2020 achieving a 30.0% response rate (*N* = 1523). Only responses from police officers (*N* = 626) were used in this article (the response rate for police officers was 23.4%). The survey captured information on basic demographic characteristics, rank and role within the police, personal vulnerabilities and caring responsibilities and a questionnaire relating to respondents’ experience of policing the pandemic, including details of their working circumstances.

### Analysis

A Principal Component Analysis (PCA) was conducted to extract the themes present in the survey. 48 Likert scale questions about Hampshire Constabulary’s response to the COVID-19 pandemic were analysed using PCA with Varimax rotation. The Kaiser–Meyer–Olkin measure of the final PCA analysis of sampling adequacy was .970, which is above the recommended figure of 0.5 (Field, 2017). Bartlett’s test of sphericity was significant (η_2_ (1128) = 45204.01, *p* < .001), confirming adequacy of the sample for PCA. A total of seven components were extracted and retained: Leadership and Safety, Line Management, Communications, Belonging, Well-being, Flexible Response to COVID-19 and Personal Protective Equipment.

The Well-being component is the sole focus of the analysis reported in this article. The Well-being component [mean (M) = 3.28, standard deviation (SD) = 1.15, α = .759] comprised the mean average of the following statements rated on a 5-point Likert scale (factor loadings are shown in parenthesis after each statement):

• ‘I feel equipped to manage both personal and work demands at the moment’ (0.47)
• ‘My levels of anxiety have increased during the COVID-19 pandemic’ (−0.68)
• ‘My work has had a negative impact upon my family/friends during the COVID-19 pandemic’ (−0.77); and
• ‘My work has had a negative impact upon my health during the COVID-19 pandemic’ (−0.81).

(See Table 3 for the Likert scores recorded for these statements and other items relating to support from line management and feelings of safety in conducting police roles).

Univariate and bivariate analyses were undertaken through a series of t-tests and ANOVAs using SPSS. Well-being was listed as the dependent variable, alongside the following independent variables: age, gender, police rank and role, time working as a police officer, working hours, working from home status, personal vulnerabilities (clinically vulnerable or extremely vulnerable to coronavirus, as per NHS, 2021), presence of vulnerabilities in the household and caring responsibilities, as well as resilience, which was measured using the answer to the statement ‘Thinking about your current working circumstances, how long do you think this is something you could comfortably maintain’?

### Interviews

**Figure 1:**
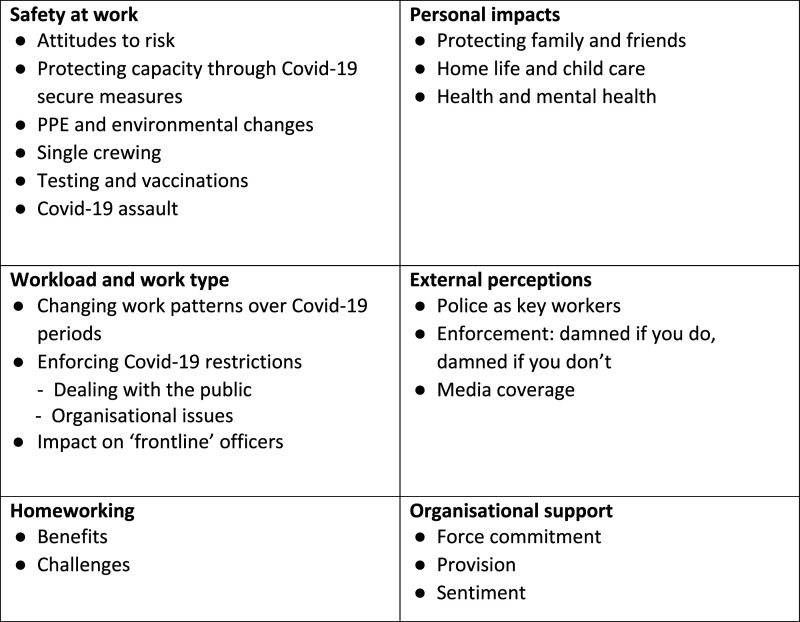
. Overarching themes and sub themes of the qualitative analysis.

Of the 271 survey respondents who were willing to participate in follow-up research, 100 were randomly selected (stratified by gender, role and rank) resulting in 39 officers being interviewed between November 2020 and February 2021 (see Table 1). Interviews were semi-structured covering the themes: changes to role during COVID-19; experience of policing the restrictions; organisational support; personal impacts; public compliance and changes in crime and policing. Given the COVID-19 restrictions, interviews were conducted via Zoom or by telephone and then transcribed. Thematic analysis was performed in NVivo (v12), broadly following the six-phase approach of Braun and Clarke (2006). Each transcript was reviewed to ensure familiarity with the overall content and the main issues raised. This was followed by an iterative process of coding the data, producing groups and hierarchies of codes and identifying emerging themes. A final review to check for consistency of coding was performed, before the overarching themes were named and defined (see Figure 1).

**Table 1.** Summary of survey respondents and participants in the qualitative interviews.

	Survey respondents (*n*=)	Interviewees (*n*=)
Rank		
PCSO	0	3
Constable	408	20
Sergeant	134	7
Inspector	50	6
Chief Inspector	18	1
Superintendent	10	2
Total	620	39
Role		
Response and Patrol	119	11
Neighbourhood policing	93	8
Investigations	121	6
Contact management	25	3
Public protection	35	3
Other (including custody, multi-agency safeguarding, offender management, etc.)	230	8
Total	623	39
Able to work from home		
No	297	22
Yes (at least partially)	319	17
Total	616	39
Sex		
Male	385	23
Female	216	16
Total	601	39
Ethnic background		
White (English, Welsh, Scottish, Irish, and British)	553	37
Mixed—White/Asian	3	1
Mixed—White/Black	15	1
Total	571	39

The survey and interviews were conducted at different periods during 2020/2021, the latter offering a longer-term perspective of policing during the pandemic than the former. The views of police officers may, of course, have changed since the research was undertaken.

## Findings

Table 2 shows the results of a multivariate analysis of the relationship between different variables and officers’ well-being, recorded in the survey. Significant associations were found for ‘current role’, ‘workload change’ and ‘caring responsibilities’, which are discussed below. Officers’ levels of ‘resilience’ had the strongest relationship with Well-being (η_2_ = .109). Respondents who felt that their current working conditions were already not sustainable (13.3%) had the lowest Well-being score (mean (M) = 2.57, SD) = .92); those who felt they could maintain their working conditions indefinitely (64.6%) had the highest Well-being score (M = 5.75, SD = 2.10 ). At the time of the survey, 14.9% of officers indicated that working during the COVID-19 pandemic had increased their likelihood leaving the police service (see Table 3).

**Table 2.** Key variables by Well-being score (results of multivariate analyses).

Independent variable (significant findings)	*M*	*SD.*	*t*	*F*	*p*	*d/*η_2_
Caring responsibilities			2.95		.003	0.24
Presence of caring responsibilities	3.11	0.83				
No caring responsibilities	3.32	0.90				
Current role				2.96	.001	.049
Neighbourhood policing	2.94	1.01				
Response and patrol	2.96	0.97				
Contact management	3.44	0.81				
Custody	2.80	0.84				
JOU roads	3.20	0.71				
Intelligence	3.13	0.58				
Investigations	3.43	0.79				
Public Protection Department	3.29	0.66				
Learning and Professional Development	3.01	0.40				
Other	3.47	0.72				
Workload change				17.08	<.001	.055
Increased	2.78	0.82				
Decreased	3.27	0.86				
Stayed the same	3.25	0.83				
Resilience—sustainability of working conditions				15.17	<.001	.109
Not sustainable now	2.57	0.92				
A few weeks	2.77	0.80				
About a month	2.91	0.74				
Two months	3.10	0.84				
Three or four months	3.03	0.83				
Indefinitely	3.36	0.79				
Independent variable (non-significant findings)						
Working hours			1.39		.164	
Gender				1.22	.295	
Age				0.95	.448	
Rank				2.16	.072	
Time working as police officer				2.15	.073	
Working from home				2.57	.053	
Personal vulnerabilities				.65	.522	
Household vulnerabilities				.92	.398

**Table 3.** Responses to well-being-related survey questions.

Survey questions		Strongly agree		Agree		Neutral		Disagree		Strongly disagree	
During the pandemic	*N*	*n*	%	*n*	%	*N*	%	*n*	%	*n*	%
I have felt safe conducting my usual role	626	68	10.9	231	36.9	132	21.1	123	19.6	72	11.5
I have felt unsafe dealing with the public	485	23	4.7	140	28.9	138	28.5	161	33.2	23	4.7
I have felt more anxious when responding to standard emergency calls compared to before	483	39	8.1	123	25.5	214	44.3	80	16.6	27	5.6
I have been abused/threatened by a member of the public	485	47	9.7	123	25.4	65	13.4	166	34.2	84	17.3
I feel equipped to manage both personal and work demands at the moment*	626	59	9.4	321	51.3	136	21.7	76	12.1	34	5.4
We are adapting well to changes in work conditions	626	65	10.4	331	52.9	125	20.0	77	12.3	28	4.5
My work has had a negative impact upon my health*	626	41	6.5	117	18.7	142	22.7	237	37.9	89	14.2
My levels of anxiety have increased*	626	77	12.3	217	34.7	128	20.4	148	23.6	56	8.9
My work has had a negative impact upon my family/friends*	626	66	10.5	156	24.9	136	21.7	205	32.7	63	10.1
I know who to talk to if I need support	626	82	13.1	373	59.6	95	15.2	49	7.8	27	4.3
My line manager is regularly checking how I am	626	140	22.4	276	44.1	104	16.6	75	12.0	31	5.0
Working has increased the likelihood of my leaving the police service	626	35	5.6	58	9.3	162	25.9	213	34.0	158	25.2

Questions with * are included in the Well-being component

Further findings from both the survey and the qualitative interviews are discussed under the following themes: safety at work; workload and work-type; homeworking; personal impacts; external perceptions of police and organisational support.

### Safety at work

All ‘frontline’ police officers interviewed described attitudes of stoic acceptance, pride and a sense of duty in facing risk and danger in their role. The underlying motivation to protect the public and ‘run towards danger’ invokes a fundamentally more complex consideration of safety in the workplace from other professions which, faced with a pandemic, can focus exclusively on minimising risks to the workforce.

‘We can't ignore what's going on; we can't barricade ourselves into our homes because we're out there to protect people... You sign up to the job to take risks’. [32]

Officers of all ranks and roles recognised the critical need to protect the organisation’s capacity to police, and the potential for high rates of sickness (through contracting COVID-19) to diminish capacity. This necessitated a new formulation of the type and loci of risk, extending from solely the external (e.g. dealing with suspects) to internal working practices designed to keep officers safe from each other.

‘What has changed most in my role? I suppose it’s the risk assessment every time we do a job, we’re now having to think of that, first from our own personal health, rather than worrying about whether our suspects that we’re going to see have a history of violence or weapons etc’. [6]

The extended arena of risk was reflected in a sizeable minority of survey respondents: 33.6% reported feeling unsafe dealing with the public; 33.5% were more anxious when responding to standard emergency calls compared to before the start of the pandemic and 31.2% felt unsafe conducting their usual role (see Table 3).

### PPE and the covid-secure workplace

All interviewees acknowledged that the implementation of covid-secure measures (changes to the office environment to facilitate social distancing, cleaning and personal hygiene practices, etc.) and the availability of Personal Protective Equipment was slow, gathering pace several months after the onset of the pandemic. Some officers were concerned by these delays and their potential exposure to the COVID-19 virus as a result. Others questioned the necessity for such extensive protective measures, pointing to perceived detrimental effects on morale and ‘team spirit’, as well as some of the practical limitations to their effectiveness.

‘I found social distancing all very difficult. I think in the beginning stages it was really quite hard and people would basically stand back and not go anywhere near you. And I think again from the work that we do and the kind of people we are we've always been fairly tactile and fairly close working and that's quite difficult to change’. [9]

### Single crewing

The majority of frontline officers interviewed (though not all) voiced significant concerns about the policy to deploy single crewed wherever possible, adopted at the beginning of the pandemic. The potential increased risk to officer safety was perceived to outweigh any reduction in the risk of contracting COVID-19.

‘Pretty much every single officer that I know and I’ve spoken to would much rather run the risk of contracting Covid from being double crewed than from being single crewed and sent into something dangerous’. [18]

Officers described feeling isolated and missed the opportunity to diffuse, unwind and reflect with a colleague, particularly after traumatic or professionally challenging incidents. Some officers felt that single crewing was merely a performative display to assuage public criticism, and questioned the policy’s actual effectiveness when officers were frequently required to come into close proximity to each other, members of the public or other emergency workers upon arrival at an incident. However, a minority of officers interviewed were supportive of single crewing, proclaiming it as a proportionate measure to mitigate the risk of officers contracting COVID-19.

### Testing, vaccinations and policy on officers attending work with sickness

The inability to test officers and police staff for COVID-19 was a source of frustration for some officers, particularly in the early stages of the pandemic. This was perceived to have undermined efforts to prevent the spread of the virus amongst the workforce. Three officers recounted cases of colleagues attending work whilst unwell and, in the absence of testing, there was little clear guidance on what threshold had to be reached before they were sent home.

Six officers were highly critical of the failure to prioritise police officers for COVID-19 vaccinations, given their daily contact with the public and potential to be ‘super spreaders’.

### Covid assault

35.1% of survey respondents reported having been abused or threatened by a member of the public during the COVID-19 pandemic (see Table 3). Five interviewees reported having been spat at by suspects or members of the public. One response and patrol officer estimated that half of her team had suffered some form of ‘COVID-19 assault’ (been spat on or coughed at) since the beginning of March 2020.

‘About a week ago I was assaulted by someone coughing at me saying they’ve got Covid, and I think that’s happened to a lot of people. It’s just almost been weaponised’. [11]

### Workload and work-type

The survey of police officers found the majority felt equipped to manage both personal and work demands during the pandemic (60.7%) and were adapting well to the changes in working conditions (63.2%) (see Table 3). Many frontline officers interviewed reported an initial drop in demand for service during the first lockdown with the benefits of being able to clear back-logs and having more time for patrolling and engaging with the public.

However, these benefits appeared to be short-lived as demand (and deployment patterns) returned to normal over the summer with the additional responsibility on frontline officers to police the COVID-19 restrictions. The survey found a significant relationship (*p* < .001) between officers experiencing an increase in workload and lower Well-being scores (see Table 2).

### Enforcing COVID-19 restrictions

Response and patrol officers, and their supervisors, both reported stresses arising from having to implement changes to restrictions frequently just hours after being announced by the Government. Some concerns were raised about officers’ enforcement actions been scrutinised and attracting criticism:

‘At the best of times without Covid, there's so much, I really pity an officer, because tomorrow morning after they’ve dealt with that, the bosses will be saying, oh why didn't they deal with it this way… but they are making that almost split decision there and then’. [12]

Some officers expressed uneasiness about the nature and legitimacy of their new role in policing COVID-19 restrictions, questioning their sense of policing purpose and their relationship with the public. Others voiced disappointment that policing restrictions had detracted from their ability to fulfil traditional police roles in tackling crime and safeguarding.

‘That doesn’t feel right because ingrained in us and what we do… and the reason why we do this job is we're here to help people and actually [it] just feels wrong to be focusing on something that inherently isn't wrong by kids going to play in a park, but actually we're not dealing with an actual crime’. [9]

### The burden on the frontline

The interviews revealed a clear sense that the burden of extra workload over the COVID-19 period was felt disproportionately by frontline officers. In the survey, the lowest average Well-being scores were recorded for custody staff (M = 2.80; SD = 0.84), neighbourhood police (M = 2.94; SD = 1.01) and response and patrol officers (M = 2.96, SD = .97) (see Table 2).

‘Resources should have been used to help response and neighbourhoods. Whereas response and neighbourhoods officers were constantly at work and then when you’re hearing other officers are working from home and getting a bit of downtime, it kind of got your back up a bit’. [29]

Several interviewees reported an increased burden resulting from colleagues being absent through sickness or having to isolate. Others identified increased stress arising from having to implement new systems at speed with no additional budget or staff resources, for example, Virtual Remand Hearings.

### Home working

Just over half of the survey respondents and just under half of the interviewees were able – at least partially – to work from home at the time the survey or interview was administered (see Table 1). The research identified both benefits and challenges arising from home working.

### Benefits of home working

Numerous benefits of working from home were reported in the interviews. The most common were improved mental health/reduced stress; greater productivity and efficiency, including reduced time and cost of travelling; better work–life balance; and increased flexibility to manage childcare and other caring or domestic responsibilities.

Officers indicated that prior to the pandemic, the organisational culture was suspicious of home working. For many respondents, being trusted to work from home was itself a sign of a healthier working environment, albeit fortuitously arrived at.

‘I would say that Hampshire Constabulary have definitely realised that they can have people work from home and get the job done…I think there was a… mistrust and reluctance to trust staff at home, will they actually get some work done?’. [5]

### Challenges arising from home working

A number of challenges arising from home working, with implications for officers’ well-being, were also identified.

#### Perceptions of unfairness

Some officers expressed frustration that the benefits of home working, in particular increased flexibility, were not universally available to all officers. As well as frontline officers, some respondents reported that a lack of IT had prevented them from being able to undertake their role at home.

#### Isolation and maintaining contact with colleagues

Many officers acknowledged the potential for isolation resulting from working from home, and the consequences this had on morale, for feeling connected to the team, and for receiving adequate supervision and line management.

‘I've also been really grateful for the fact that I can go into work because, you know, for a lot of people working from home over a long period of time is extremely hard work… I think for a lot of police officers we're interactive people, we're social people, we thrive on that banter and we thrive on being in those kind of situations and actually to have them stopped is really quite tricky’. [9]

Some officers in managerial roles reported taking deliberate steps to foster contact between team members working from home, for example, by introducing daily morning team meetings or virtual coffee meetings.

#### Facilities to work from home

Several officers interviewed talked about their frustration with IT and difficulties in accessing technical support remotely, and the impact this had on their levels of stress and anxiety. Others recognised the difficulty in finding space to work at home and the problems in sharing broadband with other family members, particularly when children were home schooling. A number of officers disclosed concerns over ‘screen fatigue’, eye strain, back problems arising from poor seating arrangements and the tendency to become less physically active as a result of not leaving the home.

### Managing workload and separating work from home life

Officers described the potential for taking on increased workload, for example, by attending successive online meetings which – when not working from home – would have been punctuated with informal social interaction, a coffee break or the necessity to travel. Several interviewees described their efforts (and in some cases failures) to retain a clear divide between their home and work lives.

‘I think trying to get that balance between your home life and your work life sometimes is tricky because you're in your house, you don't have that switch off time and you literally can walk out of the room into the kids and you're not really ready for, what's for tea?’ [10]

### Home working and shielding

Three officers described the additional difficulties of working from home as a consequence of having to shield because of personal vulnerabilities or as a result of long-term sickness. All three were response and patrol officers, and each reported feelings of guilt at not being able to support their colleagues and a sense of being unproductive.

### Personal impacts

Whilst just over half of the survey respondents (52.1%) reported that their work had no adverse effects on their health, a quarter of respondents (25.2%) experienced a negative impact of the pandemic on their health. Almost half (47.0%) reported an increase in their levels of anxiety (see Table 3).

### Anxiety about exposing family members to COVID-19

Roughly half of the officers working from police premises reported feeling anxious about contracting COVID-19 as a result of their policing duties and potentially infecting, sometimes vulnerable, family members. They described taking precautions to protect family members such as showering either at work or immediately upon returning home, washing clothes and cleaning protective equipment. Two officers spoke of their attempts to alleviate the heightened concerns of their partners or children.

‘The eldest who’s six was definitely worried about us, yeah, definitely concerned but I tried to minimise it, oh daddy’s just going to go and play hide and seek today. [5]

### Effects on home life

Whilst acknowledging the benefits of home working, many officers experienced difficulties juggling childcare and family responsibilities with work. Some officers reported having to use all of their annual leave simply to provide childcare.

‘I put myself under a lot of pressure to home-school my children at the same time as working. And bearing in mind the subject matter that I’m discussing day-in, day out, it was shut doors and ignoring my children for hours on end every day and expecting them to almost home-school themselves or using Alexa. [31]

The survey found that officers with caring responsibilities scored significantly lower on Well-being (M = 3.11, SD = .83) compared to those with no caring responsibilities (M = 3.32, SD = .90, *p* = .003) (see Table 2).

### Escaping work, recharging and mental health

Many officers revealed their frustration with not being able to take a meaningful break from work because of COVID-19 restrictions. Whilst the same grievance extends beyond policing, the absence of social and familial networks was felt to be particularly onerous given the often traumatic and stressful nature of police work.

‘So, it’s quite hard in particularly when you just need a little bit of support and when you’re doing the job we’re doing… you know like, I’ve been to some horrible things in the last few months, so actually I could have just done with a roast dinner at my mum’s and things would have been alright’. [37]

### Impacts of catching COVID-19

Four interviewees had contracted COVID-19 with two reporting severe and long-lasting health problems including fatigue, breathing difficulties and a deterioration in general health and well-being. At the time of interview, one officer had still been unable to return to frontline duty, some 9 months later.

### External perceptions of police

To varying degrees, nearly all officers reported positive public sentiment at the outset of the pandemic and a sense of ‘being swept along’ with the goodwill expressed towards the NHS.

‘People were very pleased to see us, we got claps and thumbs up on our daily patrols and it seems as if it was quite surreal’. [3]

However, most officers interviewed felt that such public goodwill had eroded as the pandemic continued into the latter part of 2020.

### Enforcement: damned if you do, damned if you don’t

Many frontline officers expressed unease with the competing public demands to both enforce restrictions, to exercise discretion with restrictions and to continue policing non COVID-19-related incidents. Many felt this placed them in a ‘no win’ situation with increasing numbers of the public holding negative views about the police.

‘I think one of the risks that we run is actually losing the support of certain sections of the public… in terms of what we're doing and what we're fundamentally meant to be doing, or what people think we're meant to be doing, by not responding to certain levels of crime’. [9]

‘I think as a result a lot of people have lost a bit of faith and trust and respect for the police because they are seeing us as kind of, you know, being those jackbooted individuals that are trying to restrict their freedoms and enforce what they see as anti-democratic legislation and restriction of personal freedoms and things’. [18]

The requirement to police organised protests, including Black Lives Matter and anti-lockdown events, heightened these concerns.

### Media coverage and public perceptions

The majority of officers interviewed remarked that both mainstream and social media distorted the reality of policing during the pandemic, fostering negative public sentiment, leaving officers demoralised and ‘on the back foot’.

‘The bad press, obviously, people only remember the bad things. They don’t remember some of the positive sides of things when we’re splitting up raves where there’s two or 300 people, or big gatherings of 50, 60 people at addresses… The thing is the media though, they generally only focus on the negative as opposed to the positive side of things. Probably it’s more of a headline grabber’. [34]

One response and patrol officer suggested that the possibility of her actions becoming headline news had left her reluctant to enforce restrictions at all.

### Organisational support

The majority of officers interviewed felt that Hampshire Constabulary’s welfare support services for staff were exemplary, with several applauding the commitment from senior ranks in the force. Many officers pointed to the low sickness rates amongst Hampshire Constabulary as evidence of effective welfare and support provision.

‘I think they've been incredible and I definitely think the welfare of all staff, whether it's officers or staff, has been at the front, and that's been really clear from the chief officer group, they have not wanted people struggling’. [10]

Other officers were keen to highlight the informal emotional and mental health support made available from colleagues and immediate supervisors, rather than the established programmes or services made available at the force level. For some, this was manifested in response to particular individual circumstances, such as being permitted time off when a family member died, and flexibility in responding to challenging family arrangements. Most survey respondents believed that they knew where to get support regarding the pandemic if they needed to (72.7%) and reported that their line manager had been regularly in touch with them (66.5%) (see Table 3).

A minority of officers were dismissive of the formal channels of support made available by the organisation; others felt that senior leaders had failed to show appreciation for their efforts during the pandemic.

## Discussion and conclusion

This study adds to the small but growing literature demonstrating the significant impacts on well-being resulting from extended role of the police (in enforcing COVID-19 restrictions) and the need to mitigate the risk of officers contracting and spreading the virus (Crest Advisory, 2020; De Camargo, 2021; Elliott-Davies, 2021). Whilst drawing on the experiences of police officers in a single police force area, the research identifies a potent combination of both *individual* and *organisational* stressors which have rapidly emerged over just a period of months, yet the impacts of which look destined to long outlive the COVID-19 restrictions themselves.

On the individual level, policing the pandemic has extended concerns about safety at work with officers facing the novel threat of contracting the COVID-19 virus through interactions with both the public and colleagues, and spreading the virus to family and friends as well as colleagues. Approximately one-third of police officers surveyed in Hampshire Constabulary reported feeling less safe in their role during the pandemic. Nearly half suffered increased anxiety and one quarter an adverse impact on their health. The toll on well-being appears to be most acute for frontline officers and those with caring responsibilities, and is strongly associated with increases in workload. These findings echo the results of previous research (Crest Advisory, 2020; Elliott-Davies, 2021; Hesketh et al., 2017; Purba and Demou, 2019) and, concerningly, represent *additional* burdens on police whose measures of well-being compared less favourably to other professions even before the pandemic (Elliott-Davies, 2018).

Assuming that COVID-19 is indeed on the path of manageable decline, it might be anticipated that some changes which have most adversely affected individual well-being can be reversed. A return to double crewing would most likely alleviate officers’ fears for their own safety and feelings of isolation. The burden of additional workload, for example, in enforcing restrictions, can reasonably be expected to dissipate and with it some of the perceived unfairness on frontline policing roles. Officers’ anxiety over contracting COVID-19 and infecting (vulnerable) family members is likely to diminish.

Organisational challenges to police well-being resulting from the pandemic present perhaps a higher degree of complexity (given this research was conducted in a single police force area and other forces may have experienced additional or different challenges, this summary is likely to understate such complexity, especially if the reader is seeking a ‘national picture’ of the impact of COVID-19). Changes to police working practices will require careful consideration. Homeworking and greater flexibility might well be genies that many police officers would be dismayed to see rebottled. Officers felt strongly that COVID-19 had shown that homeworking was both possible and productive, offering an improved work–life balance and a welcome measure of trust between employees and their line managers. However, retaining homeworking will present new challenges to officers’ well-being, for example, in providing adequate IT support, maintaining contact with team members, guarding against ‘sustained activation’ and the encroachment of work on family life and managing perceptions of unfairness amongst officers unable to work from home.

Efforts to restore police well-being post-COVID-19 are likely to be complicated further by the different attitudes of officers to risk and interventions to protect their safety. For some, social distancing and the introduction of covid-secure measures were welcome (albeit overdue) demonstrations of the organisation's commitment to their personal safety. For others, the same measures were viewed as mere performative, ‘safety-signalling’ exercises, offering little practical protection against COVID-19, and imposed at a cost on team morale and important interpersonal relationships. That one officer’s source of reassurance is another’s source of recrimination makes questions about the retention of changes to the working environment all the more difficult to navigate.

The research has highlighted how some officers faced particular challenges to implement new working practices at short notice and with limited additional resources, for example, Virtual Remand Hearings in custody units and virtual multi-agency platforms in safeguarding teams. Further investment to secure these practices as viable and effective systems that can be deployed either on a contingent or ongoing basis would increase organisational resilience in the face of future pandemics (or waves of COVID-19) and reduce the burden on officers having to (re)implement them at short notice.

On a more fundamental level, the experience of policing the pandemic appears to have left many officers with serious questions about their own sense of policing purpose. Interviewees reported disappointment with media portrayals of police during the pandemic, and yet many recognised that the very nature of policing, and their interactions with the public, had shifted. The prospect of a revised relationship between the police and the public – a re-positioning of the ‘thin blue line’ to protect public health at the expense of civil liberties – raises critical questions about police officers’ own sense of legitimacy, with important implications for their well-being, morale and raison d'être.

Police officers work in an environment of largely tacit public support but often vocal criticism. The death of Sarah Everard in March 2021 and the criticism of the police handling of subsequent protests about female safety have reminded the police that hard fought public support can be highly precarious. The findings in this research that some officers feel increasingly under attack from all quarters (‘damned if you do, damned if you don’t’) has important implications for officers’ motivation to remain within policing. As above, the possibility exists that such concerns will diminish as the need to enforce COVID-19 restrictions eases (although the timescale for a ‘return to normal’ is far from certain). However, the potential for the extra-ordinary policing required during the COVID-19 era to leave a legacy of increased public mistrust of ‘ordinary’ policing cannot be discounted.

Whilst the majority of officers were aware of sources of organisational support, one in seven reported that the experience of policing the pandemic had left them more likely to leave the profession.

Whilst, at the time of writing, the COVID-19 pandemic appears to be in retreat (at least in the UK) with increasing numbers of the population receiving vaccinations, questions remain as to whether the changes in policing that have occurred over the last 12 months will continue, either out of necessity (e.g. a future wave of the pandemic) or through choice.

The challenge facing individual police forces and UK policing in general is how to respond. Many of the larger issues are outside the direct control of the police themselves: the longevity of COVID-19 itself, the requirement to enforce virus-containing restrictions and the broader social, political and economic context into which we emerge. Yet, even within the confines of measures that police might be said to have some level of control over, lurk real navigational challenges. It is clear from this research that impacts on police well-being have not been felt evenly across the workforce, with frontline officers and those with caring responsibilities appearing to have been particularly adversely affected. While the task of re-establishing public trust and confidence in policing in the post-COVID-19 era must be a priority, legitimacy runs in multiple directions. A concentration upon police officers’ sense of their own self-legitimacy must be a part of that process. The task of ‘*repairing*’ well-being before and then moving to a focus upon *enhancing* well-being will, however, require detailed and sensitive consideration involving genuine efforts to hear the voices of those who have endured this prolonged tour of duty.
